# Factors Affecting Initiation and Retention of Medication-Assisted Recovery (MAR) within a Pilot Pharmacist-Involved Practice Model at a Federally Qualified Healthcare Center (FQHC) during the COVID-19 Pandemic

**DOI:** 10.3390/healthcare11101393

**Published:** 2023-05-11

**Authors:** Tiffany Nguyen, Thomas Craig Cheetham, Souhiela Fawaz, Richard Beuttler, Sharon Xavioer

**Affiliations:** 1 Department of Pharmacy Administration, Institute for Health Equity, AltaMed Health Services, Los Angeles, CA 90040, USA; tiffnguyen@altamed.org; 2 Department of Pharmacy Practice, Chapman University School of Pharmacy, Irvine, CA 92618, USA; 3 Chapman University School of Pharmacy, Irvine, CA 92618, USA; cheetham@chapman.edu (T.C.C.); rbeuttle@chapman.edu (R.B.); 4 Department of Biomedical and Pharmaceutical Sciences, Chapman University School of Pharmacy, Irvine, CA 92618, USA; sfawaz@chapman.edu; 5 HIV Services, AltaMed Health Services, Anaheim, CA 92801, USA

**Keywords:** Medication-Assisted Treatment, buprenorphine, MAT program, initiation, retention, Medication-Assisted Recovery, opioid use disorder, opioid dependence, team-based care

## Abstract

Background: During the COVID-19 pandemic, opioid-related overdose deaths increased. Although Medication-Assisted Treatment or Recovery (MAT or MAR) is available, initiation and retention rates vary. The goal of this study was to evaluate clinical, demographic, and Social Determinant of Health factors affecting MAR initiation, on-time initiation of medications, and successful retention in the program. The secondary goal was to evaluate the impact of a novel interprofessional practice model incorporating pharmacists. Methods: A retrospective analysis was conducted using electronic health record data from a pilot MAR Program initiated within a California Federally Qualified Healthcare Center. Results: From September 2019 to August 2020, 48 patients enrolled into the program. On-time initiation of medications occurred in 68% of patients and average program retention was 96.4 ± 95.8 days. Patients currently using opioids (*p* = 0.005) and those receiving supportive medications (*p* = 0.049) had lower odds of on-time MAR initiation. There were no statistically significant factors associated with successful retention in the program. The number of visits with members of the interprofessional team did not significantly affect on-time initiation or successful retention. Conclusions: Current opioid use and receipt of supportive medications were associated with lower on-time medication initiation. Further studies are warranted to explore additional factors which may affect initiation and retention.

## 1. Introduction

Opioid overdose deaths continue to increase, with the most common cause being synthetic opioids such as fentanyl [1]. During the COVID-19 pandemic, the number of opioid overdose deaths in the United States hit a record of 93,000 in 2020, a 29% increase from 2019 [2]. In California, there were 3244 opioid overdose deaths in 2019, with 1603 related to fentanyl [3,4]. This increased to 5363 opioid overdose deaths, with 3857 due to fentanyl in 2020.

Treatment for opioid use disorder (OUD) is available but few individuals initiate therapy and of those who initiate therapy, many have low retention [5]. Medication-Assisted Treatment (MAT) for opioid use disorder involves medication administration (buprenorphine, naltrexone, or methadone) combined with behavioral health services. Medication-Assisted Recovery (MAR) is similar to MAT, wherein medications are used for the treatment of opioid use disorder along with other psychosocial and supportive interventions; however, this terminology is finding preference in the recovery community as it may be less stigmatizing to those seeking care [6,7]. The study program will herein be referred to as a MAR program. Medication treatment has been shown to reduce mortality rates and increase the chances of recovery from opioid abuse [8]. Studies have also shown that retaining treatment is important to improve health outcomes for individuals with OUD such as prevention of return to substance use and reducing overdose risk [9]. There are, however, barriers that patients face in obtaining and maintaining proper care and treatment. Social determinants of health (social, environmental and economic factors) are among the barriers that can affect patient outcomes [10]; and underserved populations are often at the highest risk of experiencing these barriers leading to health disparities in the management of opioid use disorder. These social determinants can significantly impact the initiation and retention of MAR. 

Previous work exploring treatment pre-pandemic has determined that many factors can affect treatment initiation and utilization [11]. Secondly, it was determined that more research should be conducted in specific populations to identify barriers and facilitators for treatment. Studies on retention indicate that patients typically retain treatment for 3 months, but retention rates dramatically decrease after that period of time [12]. In addition, studies on different practice models for MAR programs have explored the involvement of various health professionals [13]. One study analyzed the effect of a physician-pharmacist collaborative practice for opioid-dependent patients and found that retention rates increased, and patients were less likely to return to use [14]. Recent studies during the COVID-19 pandemic explored prescription rates of medications such as buprenorphine and found that although there was an initial increase in the number of patients prescribed treatment in the early period of the pandemic, monthly prescription rates increased at a lower rate compared to pre-pandemic [15]. Studies also found that telehealth visits may result in higher treatment retention compared to in-person visits [16].

Our study examines how an innovative practice model of telehealth and in-person visits with an interprofessional healthcare team impacts retention rates within a pilot MAR program. To address gaps in the literature, we explored additional demographic and social determinants of health as well as clinical and patient-specific characteristics in an ethnic minority and underserved population. The primary objective was to determine which of these factors affect initiation and retention of MAR. The secondary objective was to evaluate the impact of a practice model involving an interprofessional team of a clinical pharmacist, physician, case manager, and other staff on initiation and retention of MAR.

## 2. Materials and Methods

### 2.1. Study Design and Population

This study explored demographic, patient-specific, and clinical factors that may affect MAR initiation and retention. A retrospective analysis was conducted by analyzing data collected on patients enrolled in the pilot MAR program from 1 September 2019 to 31 August 2020. 

Data from the outpatient medical clinics of the AltaMed Health Services Corporation across Orange County and Los Angeles County were included in this study. AltaMed is a multi-service medical institution and one of the largest independent Federally Qualified Health Centers (FQHC) in the United States. Electronic health data from the EPIC^®^ health record system of the AltaMed clinics were collected by study researchers in a de-identified form. The study population included men and women 18 years and older who have been prescribed MAR therapies by an X-waivered provider and had been officially enrolled in the AltaMed MAR program for treatment of opioid or substance use disorder. Official enrollment meant that the patients completed the necessary intakes and were scheduled for an initial visit with the X-waivered provider. Sample size was not calculated as all patients who were eligible and enrolled in the program were intended to be included in the analysis. At the time of the study, any provider who is prescribing buprenorphine containing medications for the purpose of treating opioid/substance use disorder must have completed training and certification to be X-waivered as indicated by the Drug Addiction Treatment Act of 2000. Patients receiving MAR medications for pain management and patients who were pregnant during the data collection period were excluded from the study as they were referred out for specialty care per program protocol.

### 2.2. Team-Based Practice Model

At AltaMed, patients enrolled in the MAR program are only provided with prescriptions for buprenorphine or buprenorphine-naloxone. Patients requesting other MAR medications are referred out as clinically appropriate. The AltaMed MAR program consists of a referral to the MAR team which is comprised of a clinical pharmacist, case manager, and X-waivered physician. Other health professionals are consulted as needed and may include nurses, behavioral health professionals, social workers, and specialty physicians. When enrolled in the MAR program, each patient has visits with the clinical pharmacist, X-waivered physician, and case manager. These visits can be telehealth (telephone/video) or in-person. Patients are referred to the program by a primary care provider, specialist, or self-referral by walking-in or calling the clinic directly. When patients are referred, the case manager calls the patient to introduce the program and complete a referral intake. Then, the patient is scheduled to speak with the clinical pharmacist who performs the initial opioid history assessment, reviews current medications and conditions, orders laboratory work, provides medication counseling, educates on naloxone, and recommends a treatment plan to the prescribing provider. After initial intakes, the patient is scheduled for an office visit with the X-waivered physician who will conduct a final assessment and provide a prescription for buprenorphine as medically appropriate. During this visit, the provider assesses the patient’s withdrawal symptoms to consider when the patient should start their first dose of buprenorphine because a 12–72 h opioid free interval is needed prior to induction. Patients may then receive a few days’ supply of supportive medications to address these specific withdrawal symptoms such as loperamide for diarrhea, ondansetron for nausea, and hydroxyzine for anxiety. Patients follow up with the physician typically every 7–14 days as they initiate treatment in order to establish stability on the medication dose. Once in the maintenance phase and stable on the medication, patients may follow up monthly or bi-monthly. Between 1 and 3 days after each physician visit, the pharmacist calls to perform follow-up clinical assessments, resolve prescription issues, monitor side effects or withdrawals, and address any other medication-related concerns. The case manager calls the patient to schedule appointments, check on prescription status, and provide appointment reminders and information. The case manager also assists the patient in addressing social determinants of health issues such as transportation needs, housing instability, and food insecurities when possible by providing patients with local resources or submitting internal referrals to appropriate teams that can help the patients (i.e., housing referral). Other staff such as nurses or clinic staff may also assist with calling the patient to schedule appointments for other medical conditions, order transportation, or schedule lab appointments.

### 2.3. Data Analysis

Data were collected using a RedCap^®^ platform and provided to the research team in a de-identified form. Demographic information included gender, race, ethnicity, age, type of health insurance, location of MAR services, marital status, education level, and employment status. Social determinants of health (SDoH) data included patients’ responses to SDoH questions that are routinely asked per protocol during medical visits and documented in patient charts. These SDoH domains include financial resource strain, food insecurity, transportation needs, physical activity, stress, social connections, depression risk, alcohol use risk, and smoking/tobacco use risk. Patients were asked about their social determinants of health as part of the standard of care upon their enrollment at AltaMed as a patient. Some patients were either not yet asked the SDoH questions, refused to answer, or documentation of the SDoH response was not available for that patient. These occasions were marked as “unknown.” Patient-specific information included past opioid or substance use history, number of opioids/substances, history of previous MAR, and knowledge of naloxone use. Clinical information included current comorbidities, urine or serum drug screen results, and clinical opiate withdrawal scores (COWS). Medication information included the name of MAR medication prescribed (drug name, quantity, day supply, instructions for use, and pharmacy type) and whether supportive medications were prescribed. Other information included number of visits/calls with each interprofessional team member. Patients who had frequent visits with the clinical pharmacist (≥2 visits), physician (≥3 visits), case manager (≥3 visits), and other staff (≥1 visit) were considered high utilizers of visits.

The outcome variables of interest were initiation in the MAR program, on-time initiation of MAR medications, and successful retention of the MAR program. Initiation in the MAR program was measured by assessing whether a patient attends their initial appointment with the X-waivered provider. On-time initiation of medications meant that patients started their medication within 2–3 days after their visits. This time frame was chosen due to the clinical nature of the buprenorphine requiring a 12–72 h opioid-free interval prior to induction with the first dose. Retention was the length of time that the patient had been taking buprenorphine as prescribed until discontinuation of MAR occurred. A patient was considered successfully retaining the program if they were still enrolled as of the last date of data collection or if they were discharged from the program due to completing the treatment regimen (i.e., tapering off). Both on-time initiation and successful retention were measured as yes or no variables. If a patient attended their follow-up medical visits, then they were considered as having retention in the program. A patient was considered discharged (i.e., not successfully retaining treatment) from the program if they no longer wished to continue, requested a different MAR medication not offered in the program, switched providers to outside AltaMed, and/or had been lost to follow up or were unable to be reached. Statistical analysis with a significance level *p*-value < 0.05 was performed using R Project for Statistical Computing Version 4.2.1 [17]. Proportional differences of categorical variables were compared using a Fischer’s exact test.

## 3. Results

### 3.1. Baseline Characteristics 

There was a total of 48 patients enrolled in the MAR program during the data collection period. The average age of patients was 39.2 years old and 67% of patients were male. Nearly half of the study population was Hispanic (*n* = 21/48; 43.7%). Most patients were single (*n* = 28/48; 58.3%). The predominant type of insurance patients had was Medi-Cal (i.e., California Medicaid) (*n* = 43/48, 89.6%) which provides coverage for the treatment options used in this pilot program. The majority of patients (*n* = 36/48; 75%) were referred to the MAR program by a prescribing provider. The three most common comorbidities that patients had were chronic pain (*n* = 19/48; 39.6%), Hepatitis C (*n* = 15/48; 31.3%), and anxiety (*n* = 12/48; 25%) or other mental health conditions (*n* = 12/48; 25%). On average, patients who disclosed the length of time of their opioid use (*n* = 38) reported using opioids for 12.0 ± 8.7 years. The two most common reasons for opioid use were for recreational use (*n* = 27/48; 56.2%) and pain management (*n* = 17/48; 35.4%). A majority of patients had previously tried MAR before (*n* = 41/48; 85.4%) with most patients having tried buprenorphine/naloxone. Table 1 further describes the baseline characteristics of the sample population.

### 3.2. Program Initiation 

Of patients enrolled in the program, there was a total of 46 (95.8%) patients who successfully completed initial visits. Two patients did not attend their initial scheduled visits with the X-waivered provider and therefore were not considered to have initiated the program. 

### 3.3. On-Time Initiation of Medications

There was a total of 201 prescriptions written for MAR. The most common medication prescribed was buprenorphine-naloxone 8–2 mg films (87/201; 43.3%) followed by buprenorphine-naloxone 4–1 mg films (55/201; 27.4%) and buprenorphine-naloxone 2–0.5 mg films (45/201; 22.4%). Most patients received 1–2 prescriptions total (*n* = 19/48; 39.6%). The maximum number of prescriptions one patient received was 17 during the study duration. Table 2 shows dosages and formulations that were prescribed for MAR. 

Most medications were prescribed as once daily dosing (71/201; 35.3%) followed by twice daily dosing (68/201; 33.8%). The average day supply prescribed was for 16.1 ± 9.5 days. The most common daily dose prescribed was 8 mg/day (77/201; 38.3%) followed by 4 mg/day (42/201; 20.9%). The average daily dose was 9.8 mg/day. The maximum daily dose prescribed was 32 mg/day (6/201; 3.0%). Most patients did require or receive supportive medications (*n =* 35/48; 73%) to help manage withdrawal symptoms during the initiation period of buprenorphine. On average, 68.0% ± 39.6% of patients had on-time initiation of MAR medication. The most common reason for not being able to initiate the medication on time was issues with picking up the medication (including pharmacy billing issues, transportation issues, medication not in stock). Both current opioid use (*p* = 0.005) and supportive medications (*p* = 0.049) were statistically significantly associated with lower on-time initiation of MAR medications. While there were other variations in rates of on-time initiation among different groups in this study population such as gender, race, and SDoH factors, they were not found to be statistically significant. Table 3 shows the univariate analysis of different variables with on-time initiation. 

### 3.4. Successful Retention in the Program

The mean number of days retained in the program was 96.4 ± 95.8 days with a minimum of 0 days to a maximum of 371 days. At the end of the data collection period, 21/48 (43.8%) patients had successfully retained the program and were stable on treatment. Two of those patients had successfully tapered off treatment. Successful retention rates varied among groups such as gender, race, and SDoH factors, but no variables were demonstrated to be statistically significant. Table 4 shows the univariate analysis of different variables with successful retention. 

In total, 27/48 (56.2%) patients had been discharged from the program. The main reasons for discharge were being lost to follow up or inability to reach the patient after follow up (*n* = 12/29, 41.4%) and requiring a referral to external services (*n =* 11/29, 37.9%). The majority of patients who required external services requested inpatient detoxification centers or methadone clinics. 

### 3.5. Team-Based Practice Model 

On average, patients had 3.04 ± 3.49 visits with the clinical pharmacist, 4.68 ± 3.95 visits with the physician, and 6.14 ± 6.4 visits with the case manager. There were 16 patients who had an average of 0.93 ± 2.12 visits from other additional clinical/non-clinical staff including nursing, behavioral health, and case management. A total of 56.5% of patients were high utilizers of clinical pharmacy visits, 63.0% of patients for physician visits, 65.2% of patients for case manager visits, and 34.8% of patients for other staff visits. There were no statistically significant results on utilizers of interprofessional team member visits with on-time initiation or successful retention. Table 5 shows the univariate analysis of high/low utilizers of each team member visit with on-time initiation of medications and successful retention in the program. 

## 4. Discussion

On average, patients in this study retained the MAR program for 96.4 ± 95.8 days. This was comparable to a study by Timko et al. that also reported 3-month retention rates with higher rates in patients who received higher doses of medications [12]. When comparing gender, although not statistically significant, rates differed for on-time initiation and successful retention within our study population between females and males, which aligns with previous literature which showed that gender disparities exist in MAR. Haddad et al. found that gender disparities exist in buprenorphine treatment, in which females may need additional preventive health screenings and therefore may utilize more healthcare services [18]. Ober et al. also found that women may be more likely to initiate medication treatment for opioid use disorder in FQHCs [19]. Further study is warranted to better understand what drives potential differences in MAR between genders. In evaluating differences among racial groups, variations in rates of on-time initiation and successful retention occurred but these were not statistically significant. The presence of variations among racial groups aligns with previous studies which have suggested there are race-based disparities in MAR. A study by Cantone et al. reported that patients with no insurance, those with Medicaid or Medicare, older-age individuals, non-Hispanic whites, and those with psychiatric conditions or tobacco use disorder had lower odds of initiating medication treatment [20]. Anderson et al. explored racial and ethnic disparities in MAR during the COVID-19 pandemic in a Louisiana population and found similarly that disparities exist amongst Black, Hispanic, and White populations [21]. Further study is warranted in a larger population to understand the impact of any racial and ethnic disparities. It will also be important to determine whether differences in race and ethnicity occur post COVID-19 pandemic.

Studies regarding social determinants of health and opioid use have concluded that lack of stable housing, economic hardship, and social issues can lead to higher rates of opioid use disorder [22]. Based on our pilot study, we hypothesize that those with higher smoking risk, higher alcohol risk, and higher financial strain will have lower successful retention in the program and lower rates of on-time initiation. Though we did not identify these factors to be statistically significant, further study in a larger population may provide greater insight. Concurrent use of substances such as tobacco and alcohol are known to impact a patient’s health and long-term goals. McKelvey et al. found that smoking cessation can have more positive effects on substance use outcomes [23]. Our study identifies that future research may be needed to identify how cessation of smoking and alcohol may affect retention for opioid dependence treatment. Difficulty paying for medications due to financial strain may also be a cause of difficulties to treatment adherence. Different insurers provide different levels of coverage which may further cause barriers in payment and accessibility to treatment. In Canada, a study involving the effects of SDoH on young adults showed similarly that financial problems affected initiation and retention [24]. Present food insecurities and high stress levels showed lower success rates and lower rates of on-time initiation. Patients may be choosing between having food to survive and paying for medications, which can affect their overall retention in a program as well as ability to adhere to and pick up medications. Similarly, a study by Hooker et al. found that there was lower retention when there were mental health symptoms, low income, food insecurities, and lack of transportation [25]. However, in their study, the population was predominantly Caucasian (75%) with 7.8% Latino participants, and our study included a larger Latino population (33.3%). 

Social connections play an important role in mental health and daily life. MAR medications such as buprenorphine can have an impact on social connections by increasing patient performance with daily life activities and satisfaction [26]. Patients in this study who had a slight or moderate level of isolation might have lower on-time initiation of medications. This warrants further studies as social connectedness, especially during the COVID-19 era when patients may not have been permitted to leave their homes or attend in-person recovery groups, may impact a patient’s recovery environment and support system. Although our results for demographics and SDoH did not show statistical significance, our study indicated that these variables should be explored further with a larger study population.

In this study, previous MAR use did not impact success in the program. Patients who were not successful on previous MAR medications might have lower motivation, which can impact retention. More studies related to reasons for discontinuing previous MAR and a patient’s motivation level are important as shown from these results. Based on the results of this study, we hypothesize that participants who reported multiple opioid use could have slightly higher success than those who only reported single opioid use. This might be due to increased motivation from the patient’s perspective and urgency to seek treatment due to higher risks when combining multiple opioids and substances. Providers may also be more aggressive in treatment regimens when managing patients who take multiple opioids due to higher perceived risks. While successful retention rates for patients with multiple substance use (in addition to opioids) were lower, they were not statistically significant. We hypothesize that this may continue to hold true with a larger population, as these individuals are often referred out to receive more intensive treatment as the institution’s outpatient MAR program does not treat dependence on non-opioid substances such as methamphetamines. 

Current opioid use resulted in statistically significant lower odds of on-time initiation of medications. Due to the nature of the prescribed medication in the program (buprenorphine), patients must have an opioid-free interval before induction with the first dose. Therefore, some patients may not be ready to start treatment yet as they may still be using opioids or are not able to achieve the full opioid-free interval period. Furthermore, supportive medications also resulted in statistically significant lower odds of on-time initiation of medications. However, both receiving and not receiving supportive medications showed an impact. Patients who are currently undergoing withdrawal symptoms may require supportive medications; however, due to other SDoH, such as financial strain, they may not have gone to pick up the supportive medications that were prescribed. It is important to note that during the COVID-19 pandemic, many individuals were impacted financially, and lock-down measures could result in the inability to have transportation [27,28]. This could also be what impacted these results. Additionally, supportive medications may not have been prescribed at the same time as the MAR medication. As the case manager follows up with the patient 1–2 days after the visit, the patient may note that they are experiencing withdrawals and thus the case manager would contact the prescriber to send supportive medication prescriptions to the pharmacy. Patients in this scenario may not have started their MAR medications until they receive the supportive medications. Furthermore, additional trips to the pharmacy may lead to additional delays in starting treatment. It is important to evaluate the program workflow and determine when it is optimal to prescribe supportive medications.

The pilot program explored an innovative practice model that included visits with a clinical pharmacist, physician, case manager, and other team members. Those who were high utilizers of pharmacist visits had lower on-time initiation. The pharmacist in this practice model participates in the workflow during the initial intake process for all patients and on an as-needed basis for follow up. Therefore, patients who needed more frequent follow up calls from the pharmacist may have had lower levels of motivation to start the medication or had side effects that they needed to discuss. Patients may also have faced medication access barriers, which the pharmacist had to resolve prior to the patient starting the medication. These barriers included medication not being in stock, pharmacy billing issues, and transportation issues. Other pharmacist-involved MAR models involve a pharmacist supervising the dispensing or induction of MAR medications. One study by DiPaula et al. analyzed the effect of a physician–pharmacist collaborative practice model in a buprenorphine program in a small population of 12 patients and found that such a practice model where the pharmacist provides physician guidance on appropriate dosing and tapering may be effective to increase program retention [14]. Their model is similar to ours; however, as a pilot program, we may consider revising our workflow to have more structured visits with the pharmacist to be able to better understand the impact of the pharmacist role in MAR. Additionally, our study was performed during the beginning of the COVID-19 era. We changed some of the workflow to allow telehealth visits as the regulations around prescribing buprenorphine changed. Studies regarding retention of treatment during COVID-19 showed that telehealth options resulted in better retention [16]. Increasing the sample size and extending the data collection period to the middle of the pandemic may show more information on how telehealth vs. in-person visits in our program could affect retention.

Although the practice model did not show statistically significant results, we hypothesize that retention may be important to explore further. Retention in the program among those with high utilization of pharmacist visits was comparable to visits with other team members. Patients with high utilization of physician visits also had lower on-time initiation. This could be due to patients needing closer follow up with the provider to establish stabilization of the dose or needing additional medications such as those to help with withdrawal symptoms. Patients who needed closer follow up may have been new to the MAR medication and were in the induction phase of treatment. Those who had more visits with the physician, however, did have a higher rate of successful retention in the program. This is similar to other examples in the literature in which it is known that when patients have closer follow up with their physicians, their questions or concerns are addressed, their symptoms are closely monitored to prevent adverse health events and hospitalizations, and they have overall better outcomes [29]. 

Patients who were high utilizers of case manager visits did not seem to have higher on-time initiation nor successful retention. This result is similar to that of the pharmacist visits because the case manager may have also had to follow up with the patient more frequently if they had not started their medication, had scheduling issues or other barriers that needed to be addressed. Visits with other team members were similar across on-time initiation and retention; however, the impact of other team members is not yet clear because there were various individuals who may have assisted patients during the program based on their individual needs. Team members such as nurses and behavioral health clinicians may be involved in a patient’s care and further studies should explore their roles in this practice model. Although the results did not demonstrate a statistically significant impact on on-time initiation of medications or successful retention, they do indicate that there is potential for the different visit types with team members to affect initiation or retention and this warrants future studies.

### Strengths and Limitations 

The strengths of this study include exploration of different variables such as social determinants of health that had not been studied before. The study involved an innovative pilot MAR program with multiple interprofessional team members. As a result of the pilot nature of the program, the sample size was based on enrollment dates and no power calculation was conducted. Due to the study method of a retrospective chart review, the data were restricted to what was documented on the electronic health record system. Not all information was documented in the same way in each patient chart as each provider may use different templates and documentation techniques. There may also be incomplete SDoH information on the charts due to patients declining to answer or not having been asked the SDoH screening question because the SDoH questionnaire workflow is dependent upon each clinic. Because data were limited to only what was available in the health record, data and information regarding consultations (informal) between the pharmacist and physician or other team members could not be collected. Data regarding reasons why specific doses of buprenorphine were used for individual patients were not available. This is a limitation, because underdosing based on substances patients may be using can lead to lower treatment efficacy. 

## 5. Conclusions

There are many reasons why patients delay initiating prescribed MAR regimens and have trouble with treatment retention. We found that supportive medications and current opioid use resulted in lower on-time initiation of medications. Although other factors did not show statistical significance, our study is from a pilot program and we hypothesize that these factors may affect patient outcomes in a MAR program. Many SDoH factors can affect patient outcomes and therefore it is important to understand these more clearly and develop specific roles for each interprofessional team member that can help address these barriers. It may also be important to explore what factors contribute to reasons for not starting a medication on time. These could be patient-specific such as those at the motivation level or external barriers such as access issues. An interprofessional team may have an effect on increased retention; however, further research in a larger population and post-pandemic may provide greater insight. It may also be important to evaluate what methods, in the future, could be offered to improve motivation to initiate and retain MAR. 

## Figures and Tables

**Table 1:** Baseline Characteristics (*N* = 48).

Age	39.2 ± 12.0 years		
Gender		Health Insurance	
Male	32 (66.7%)	Medi-Cal (Medicaid)	43 (89.6%)
Female	16 (33.3%)	Un-Insured	4 (8.3%)
		Private Insurance	1 (4.2%)
Race		Marital Status	
White	29 (60.4%)	Single	28 (58.3%)
Latino	16 (33.3%)	Married	5 (10.4%)
Asian	2 (4.2%)	Living with Partner	3 (6.3%)
Black	1 (2.2%)	Separated/Divorced	9 (18.8%)
		Other	3 (6.3%)
Ethnicity		Referral source to MAR Program	
Non-Hispanic	26 (56.3%)	Referred by provider	36 (75.0%)
Hispanic	22 (42.7%)	Referred by health plan	4 (8.3%)
		Self-discovered/walk-in	8 (16.7%)
Comorbid Medical Conditions *		Reasons for using opioids *	
Pain	26 (54.2%)	Pain management	24 (50.0%)
Chronic Pain	19 (39.6%)	Recreational use	35 (72.9%)
Other Pain	7 (14.6%)	Prevent withdrawals	3 (6.3%)
Hepatitis C	15 (31.3%)	Depression/Anxiety	1 (2.2%)
Mental Health	31 (64.6%)	Did not disclose	4 (8.3%)
Depression	7 (14.6%)		
Anxiety	12 (25.0%)		
Other	12 (25.0%)		
Diabetes	2 (4.2%)		
Hypertension/Cardiac	9 (18.8%)		
Hematologic Condition	3 (6.3%)		
Other Chronic Disease	8 (16.7%)		
Previous Use of MAR		County Receiving MAR Services	
Yes	41 (85.4%)	Orange County	38 (79.2%)
No	6 (12.5%)	Los Angeles County	10 (20.8%)
Did not disclose	1 (2.2%)		

* Patients could have more than one comorbid medical condition and reason for using opioids.

**Table 2:** MAR Medications Prescribed.

Medication Name	Total of Prescriptions
Buprenorphine-naloxone 2–0.5 mg Films	45
Buprenorphine-naloxone 4–1 mg Films	55
Buprenorphine-naloxone 8–2 mg Films	87
Buprenorphine-naloxone 0.7/0.18 mg Tablets	4
Buprenorphine-naloxone 5.7/1.4 mg Tablets	6
Buprenorphine 8 mg Tablets	4
**Total MAR Prescriptions**	201

**Table 3:** Fischer’s Exact Test analysis of independent variables with on-time initiation *.

Variable	No: *n* (%)	Yes: *n* (%)	*p*-Value
Gender (*n* = 48)			
Female (*n* = 16)	11 (68.8)	5 (31.2)	*p* = 0.535
Male (*n* = 32)	18 (56.2)	14 (43.8)	
Ethnicity (*n* = 48)			
Non-Hispanic (*n* = 26)	15 (57.7)	11 (42.3)	*p* = 0.771
Hispanic (*n* = 22)	14 (63.6)	8 (36.4)	
Race (*n* = 48)			
White (*n* = 29)	20 (69.0)	9 (31.0)	*p* = 0.129
Latino (*n* = 16)	8 (50.0)	8 (50.0)	
Asian (*n* = 2)	0 (0)	2 (100.0)	
Black (*n* = 1)	1 (100.0)	0 (0)	
Insurance (*n* = 48)			
Medi-Cal (*n* = 43)	27 (62.8)	16 (37.2)	*p* = 0.424
Private Insurance (*n* = 1)	0 (0)	1 (100.0)	
Un-insured (*n* = 4)	2 (50.0)	2 (50.0)	
SDoH—Financial Strain (*n* = 30)			
High Risk (*n* = 6)	4 (66.7)	2 (33.3)	*p* = 1.000
Medium Risk (*n* = 20)	13 (65.0)	7 (35.0)	
Low Risk (*n* = 4)	3 (75.0)	1 (25.0)	
SdoH—Food Insecurity (*n* = 32)			
Not Present (*n* = 25)	15 (60.0)	10 (40.0)	*p* = 0.374
Present (*n* = 7)	6 (85.7)	1 (14.3)	
SdoH—Transportation Needs (*n* = 34)			
Not Present (*n* = 31)	19 (61.3)	12 (38.7)	*p* = 1.000
Present (*n* = 3)	2 (66.7)	1 (33.3)	
SdoH—Physical Activity (*n* = 28)			
Inactive (*n* = 12)	10 (83.3)	2 (16.7)	*p* = 0.076
Insufficiently Active (*n* = 7)	2 (28.6)	5 (71.4)	
Sufficiently Active(*n* = 9)	6 (66.7)	3 (33.3)	
SdoH—Stress (*n* = 29)			
Not Present (*n* = 20)	12 (60.0)	8 (40.0)	*p* = 0.431
Present (*n* = 9)	7 (77.8)	2 (22.2)	
SdoH—Social Connections (*n* = 18)			
Isolated (*n* = 2)	1 (50.0)	1 (50.0)	*p* = 0.561
Moderately Isolated (*n* = 9)	7 (77.8)	2 (22.2)	
Slightly Isolated (*n* = 7)	6 (85.7)	1 (14.3)	
SdoH—Depression Risk (*n* = 38)			
High Risk (*n* = 6)	4 (66.7)	2 (33.3)	*p* = 1.000
Low Risk (*n* = 2)	1 (50.0)	1 (50.0)	
Not at Risk (*n* = 30)	17 (56.7)	13 (43.3)	
SdoH—Smoking Risk (*n* = 42)			
High Risk (*n* = 21)	14 (66.7)	7 (33.3)	*p* = 0.217
Medium Risk (*n* = 7)	2 (28.6)	5 (71.4)	
Low Risk (*n* = 14)	9 (64.3)	5 (35.7)	
SdoH—Alcohol Risk (*n* = 32)			
High Risk (*n* = 4)	3 (75.0)	1 (25.0)	*p* = 0.613
Not at Risk (*n* = 28)	15 (53.6)	13 (46.4)	
Current Opioid Use (*n* = 44)			
No (*n* = 19)	7 (36.8)	12 (63.2)	*p* = 0.005
Yes (*n* = 25)	20 (80.0)	5 (20.0)	
	Previous MAR (*n* = 48)		
No (*n* = 7)	3 (42.9)	4 (57.1)	*p* = 0.412
Yes (*n* = 41)	26 (63.4)	15 (36.6)	
Multiple Opioid Use (*n* = 48)			
No (*n* = 33)	20 (60.6)	13 (39.4)	*p* = 1.000
Yes (*n* = 15)	9 (60.0)	6 (40.0)	
Multiple Substance Use (*n* = 48)			
No (*n* = 18)	9 (50.0)	9 (50.0)	*p* = 0.362
Yes (*n* = 30)	20 (66.7)	10 (33.3)	
	Current Naloxone (*n* = 39)		
No (*n* = 28)	18 (64.3)	10 (35.7)	*p* = 0.718
Yes (*n* = 11)	6 (54.5)	5 (45.5)	
Supportive Meds Given (*n* = 48)			
No (*n* = 35)	18 (51.4)	17 (48.6)	*p* = 0.049
Yes (*n* = 13)	11 (84.6)	2 (15.4)	

* Participant counts for the independent variables differ because some patients declined to answer, were not asked the question or the information was not available in the chart during data collection.

**Table 4:** Fischer’s Exact Test analysis of independent variables with successful retention *.

Variable	No: *n* (%)	Yes: *n* (%)	*p*-Value
Gender (*n* = 48)			
Female (*n* = 16)	8 (50.0)	8 (50.0)	*p* = 0.555
Male (*n* = 32)	19 (59.4)	13 (40.6)	
Ethnicity (*n* = 48)			
Non-Hispanic (*n* = 26)	16 (61.5)	10 (38.5)	*p* = 0.561
Hispanic (*n* = 22)	11 (50.0)	11 (50.0)	
Race (*n* = 48)			
White (*n* = 29)	20 (69.0)	9 (31.0)	*p* = 0.076
Latino (*n* = 16)	6 (37.5)	10 (62.5)	
Asian (*n* = 2)	1 (50.0)	1 (50.0)	
Black (*n* = 1)	0 (0)	1 (100.0)	
Insurance (*n* = 48)			
Medi-Cal (*n* = 43)	24 (55.8)	19 (44.2)	*p* = 0.449
Private Insurance (*n* = 1)	0 (0)	1 (100.0)	
Un-insured (*n* = 4)	3 (75.0)	1 (25.0)	
SDoH—Financial Strain (*n* = 30)			
High Risk (*n* = 6)	5 (83.3)	1 (16.7)	*p* = 0.528
Medium Risk (*n* = 4)	2 (50.0)	2 (50.0)	
Low Risk (*n* = 20)	11 (55.0)	9 (45.0)	
SDoH—Food Insecurity (*n* = 32)			
Not Present (*n* = 25)	14 (56.0)	11 (44.0)	*p* = 0.211
Present (*n* = 7)	6 (85.7)	1 (14.3)	
SDoH—Transportation Needs (*n* = 34)			
Not Present (*n* = 31)	19 (61.3)	12 (38.7)	*p* = 1.000
Present (*n* = 3)	2 (66.7)	1 (33.3)	
SDoH—Physical Activity (*n* = 28)			
Inactive (*n* = 12)	9 (75.0)	3 (25.0)	*p* = 0.607
Insufficiently Active (*n* = 7)	4 (57.1)	3 (42.9)	
Sufficiently Active (*n* = 9)	5 (55.6)	4 (44.4)	
SDoH—Stress (*n* = 29)			
Not Present (*n* = 20)	11 (55.0)	9 (45.0)	*p* = 0.412
Present (*n* = 9)	7 (77.8)	2 (22.2)	
SDoH—Social Connections (*n* = 18)			
Isolated (*n* = 2)	0 (0)	2 (100.0)	*p* = 0.163
Moderately Isolated (*n* = 9)	7 (77.8)	2 (22.2)	
Slightly Isolated (*n* = 7)	5 (71.4)	2 (28.6)	
SDoH—Depression Risk (*n* = 38)			
High Risk (*n* = 6)	2 (33.3)	4 (66.7)	*p* = 0.417
Low Risk (*n* = 2)	0 (0)	2 (100.0)	
Not at Risk (*n* = 30)	16 (53.3)	14 (46.7)	
SDoH—Smoking Risk (*n* = 42)			
High Risk (*n* = 21)	11 (52.4)	10 (47.6)	*p* = 0.124
Medium Risk (*n* = 12)	9 (75.0)	5 (41.7)	
Low risk (*n* = 7)	1 (14.3)	6 (85.7)	
SDoH—Alcohol Risk (*n* = 32)			
High Risk (*n* = 4)	3 (75)	1 (25)	*p* = 0.279
Not at Risk (*n* = 28)	10 (35.7)	18 (64.3)	
Current Opioid Use (*n* = 44)			
No (*n* = 19)	7 (36.8)	12 (63.2)	*p* = 0.127
Yes (*n* = 25)	16 (64.0)	9 (36.0)	
Previous MAR (*n* = 48)			
No (*n* = 7)	3 (42.8)	4 (57.1)	*p* = 0.683
Yes (*n* = 41)	24 (58.5)	17 (41.4)	
Multiple Opioid Use (*n* = 48)			
No (*n* = 33)	21 (63.7)	12 (36.4)	*p* = 0.209
Yes (*n* = 15)	6 (40.0)	9 (60.0)	
Multiple Substance Use (*n* = 48)			
No (*n* = 18)	9 (50.0)	9 (50.0)	*p* = 0.558
Yes (*n* = 30)	18 (60.0)	12 (40.0)	
Current Naloxone (*n* = 39)			
No (*n* = 28)	19 (67.9)	9 (32.1)	*p* = 0.277
Yes (*n* = 11)	5 (45.5)	6 (54.5)	
Supportive Meds Given (*n* = 48)			
No (*n* = 35)	19 (54.3)	16 (45.7)	*p* = 0.750
Yes (*n* = 13)	8 (61.5)	5 (38.5)	

* Participant counts for the independent variables differ because some patients declined to answer, were not asked the question, or the information was not available in the chart during data collection.

**Table 5:** Fischer’s Exact Test analysis of High/Low utilizers of interprofessional team member visits with on-time initiation of MAR medications and successful retention.

Variable	No On-Time Initiation *n* (%)	Yes On-Time Initiation *n* (%)	*p*-Value On-Time Initiation	No Successful Retention *n* (%)	Yes Successful Retention *n* (%)	*p*-Value Successful Retention
Utilizer of Pharmacy Visits (*n* = 48)						
High (*n* = 24)	18 (75.0)	6 (25.0)	*p* = 0.075	14 (58.3)	10 (41.7)	*p* = 1.000
Low (*n* = 24)	11 (45.8)	13 (54.2)		13 (54.2)	11 (45.8)	
Utilizer of Physician Visits (*n* = 48)						
High (*n* = 29)	17 (58.6)	12 (41.4)	*p* = 1.000	13 (44.8)	16 (55.2)	*p* = 0.075
Low (*n* = 19)	12 (63.2)	7 (36.8)		14 (73.7)	5 (26.3)	
Utilizer of Case Manager Visits (*n* = 48)						
High (*n* = 30)	21 (70.0)	9 (30.0)	*p* = 0.127	20 (66.7)	10 (33.3)	*p* = 0.077
Low (*n* = 18)	8 (44.4)	10 (55.6)		7 (38.9)	11 (61.1)	
Utilizer of Visits with Other Team Members (*n* = 48)						
High (*n* = 16)	10 (62.5)	6 (37.5)	*p* = 1.000	8 (50.0)	8 (50.0)	*p* = 0.555
Low (*n* = 32)	19 (59.4)	13 (40.6)		19 (59.4)	13 (40.6)	

## Data Availability

Data from this study may be available upon reasonable request by emailing the corresponding author. Data are not publicly available due to privacy and confidentiality.
